# The building blocks of the full body ownership illusion

**DOI:** 10.3389/fnhum.2013.00083

**Published:** 2013-03-21

**Authors:** Antonella Maselli, Mel Slater

**Affiliations:** ^1^EVENT Lab, Facultat de Psicologia, Universitat de BarcelonaBarcelona, Spain; ^2^Institució Catalana Recerca i Estudis AvancatsBarcelona, Spain; ^3^Department of Computer Science, University College LondonLondon, UK

**Keywords:** perceptual illusions, full body ownership illusion, rubber hand illusion, out-of-body experiences, multisensory integration, first person perspective, virtual reality

## Abstract

Previous work has reported that it is not difficult to give people the illusion of ownership over an artificial body, providing a powerful tool for the investigation of the neural and cognitive mechanisms underlying body perception and self consciousness. We present an experimental study that uses immersive virtual reality (IVR) focused on identifying the perceptual building blocks of this illusion. We systematically manipulated visuotactile and visual sensorimotor contingencies, visual perspective, and the appearance of the virtual body in order to assess their relative role and mutual interaction. Consistent results from subjective reports and physiological measures showed that a first person perspective over a fake humanoid body is essential for eliciting a body ownership illusion. We found that the illusion of ownership can be generated when the virtual body has a realistic skin tone and spatially substitutes the real body seen from a first person perspective. In this case there is no need for an additional contribution of congruent visuotactile or sensorimotor cues. Additionally, we found that the processing of incongruent perceptual cues can be modulated by the level of the illusion: when the illusion is strong, incongruent cues are not experienced as incorrect. Participants exposed to asynchronous visuotactile stimulation can experience the ownership illusion and perceive touch as originating from an object seen to contact the virtual body. Analogously, when the level of realism of the virtual body is not high enough and/or when there is no spatial overlap between the two bodies, then the contribution of congruent multisensory and/or sensorimotor cues is required for evoking the illusion. On the basis of these results and inspired by findings from neurophysiological recordings in the monkey, we propose a model that accounts for many of the results reported in the literature.

## 1. Introduction

The experience of our body results from the complex interplay of various perceptual streams involving vision, touch, proprioception, interoception, motor control, and vestibular sensations. Strong evidence supporting this statement comes from a number of neurological conditions where the sense of the body becomes altered as a consequence of focal brain lesions, limb amputation or deafferentation. Examples of such conditions are somatoparaphrenia and associated anosognosia (Berlucchi and Aglioti, 1997, 2010; Vallar and Ronchi, 2009), phantom limb sensations (Giummarra et al., 2007), and out-of-body experiences (OBEs) (Blanke and Mohr, 2005).

Altered body perceptions analogous to those experienced in lesioned patients, can be temporally induced in healthy subjects in controlled experimental settings, where the delivery of anomalous sensory stimuli is systematically manipulated. These altered perceptual states are usually referred to as bodily illusions and have been extensively used for studying body perception, self-consciousness and their underlying neural correlates. In particular, experimentally controlled bodily illusions allow, to some extent, the isolation of the various components that converge in the holistic experience of our bodies.

In this paper we present a study that investigates the main perceptual components of the full body ownership illusion, a specific type of bodily illusion in which healthy subjects experience an artificial body as if it were their own physical body. This illusion is particularly interesting for the study of self-consciousness as it relies on an altered representation of the entire body (Blanke and Metzinger, 2009; Blanke, 2012) and, as such, it is complementary to the “rubber hand illusion” (RHI) paradigm in the study of body perception.

In the original version of the RHI (Botvinick and Cohen, 1998), participants see a rubber hand located in front of them in a similar posture to their corresponding but hidden real hand. Both rubber and real hands are stroked by the experimenter. When the strokes on the real and rubber hands are delivered synchronously and in the corresponding spatial locations, most participants experience the rubber hand as if it were their own and feel the touch as originating from the rubber hand itself; the same does not happen, or to a much lesser extent, when the stroking is asynchronous, or when there are other inconsistencies (such as spatial) between the stroking on both hands. A large number of studies have adapted and extended this classical set-up providing experimental evidence about the different perceptual components that contribute to the RHI. These include the spatial configuration and appearance of the artificial hand and the delivery of multisensory and/or sensorimotor stimulation. The spatial configuration of the artificial hand with respect to the real body affects the illusion. For example, the illusion does not occur when anatomical constraints are violated: when the rubber hand is located outside the participant's peripersonal space (e.g., Lloyd, 2007), when it is in impossible postures (e.g., Ehrsson et al., 2004; Tsakiris and Haggard, 2005), or when it does not represent the main topological features of a hand (Tsakiris and Haggard, 2005). In terms of perceptual cues, the spatial configuration component corresponds to a stream of visuoproprioceptive information in which the spatial location encoded by vision is compared with the one encoded by proprioception. The body's spatial location encoded by proprioception can be altered, so that it does not always coincide with the position of the physical body. This happens in RHI experiments, where the illusion is typically associated with a mislocalization of the real hand toward the fake hand. Inherent to the RHI paradigm is the delivery of visuotactile stimulation, which has to be synchronous in order to evoke the illusion. However, it has been shown that the illusion is also experienced when visuotactile stimulation is substituted by other modalities of multisensory and/or sensorimotor stimulation, e.g., with sensorimotor contingencies in active or passive movements (Tsakiris et al., 2006; Kalckert and Ehrsson, 2012). This shows that the onset of the illusion is triggered more generally from a stream of congruent multimodal bodily signals (multisensory or sensorimotor) and does not necessarily require visuotactile integration. Finally, visual appearance has been found to play a critical role in the illusion: the fake hand does not need to be necessarily realistic for the illusion to take place, as shown by the numerous reports of ownership experienced over plastic-looking hands or elongated limbs (Schaefer et al., 2007; Kilteni et al., 2012; Preston and Newport, 2012); however, higher levels of realism of the artificial hand, in terms of texture and shape, have been found to enhance the strength of the illusion (Haans et al., 2008). The overall emerging scenario is that the sense of ownership over an external object requires the convergence of two main sources of information: (1) the *on-line* driving contribution from bottom–up processing of congruent multimodal perceptual cues and (2) the modulating top–down machinery based on a flexible but still robust *prior* internal body representation that requires that some key anatomical constraints in terms of body shape and visual perspective are preserved (see Graziano and Botvinik, 2002; Makin et al., 2008; Tsakiris, 2010).

Analogous perceptual components have been identified for the illusions of owning a whole artificial body. However, the relative importance of each of these components and their mutual interaction are still a subject of debate. This is partly due to the fact that different experimental procedures have been introduced to extend the RHI for dealing with whole bodies. Two main set-ups have been used and, interestingly, the illusions evoked in the two cases were not the same. Both set-ups use a head-mounted-display (HMD) to occlude the real body from vision and to display the artificial body in stereo. In one case participants saw their own body or that of a mannequin as filmed from 2 m behind. When exposed to synchronous stroking seen on the back of the visualized body and felt on their own back, participants reported experiencing the sensation of “seeing their own perceived body from a distance” and of perceiving touch as originating from the distant body (Lenggenhager et al., 2007, 2009; Aspell et al., 2009; Ionta et al., 2011). Alternatively, when the stroking was felt on the chest and seen from a first person perspective (i.e., the stick seen as stroking the spatial location corresponding to that of the real chest), the resulting illusion was characterized as a feeling of having one's own center of awareness moved behind the seen body, to the first person perspective position from where the scene was being observed. The true body seen in front was perceived as something like an “empty shell” or a “disowned body” (Ehrsson, 2007; Lenggenhager et al., 2009; Guterstam and Ehrsson, 2012). Although the illusions experienced in these two cases are notably different, they are both referred to as experimentally induced OBEs. In a different experimental set-up, a mannequin (Petkova and Ehrsson, 2008; Petkova et al., 2011) or a virtual body (Slater et al., 2010) was displayed from a first person perspective (1PP), where the artificial body visually substituted the obscured real body. Synchronous stroking of the real and virtual bodies typically induces the illusion of owning the artificial body, which is then perceived as the origin of sensory signals like touch and vision. We refer to this as full body ownership illusion.

Two principal open issues emerge from the work so far on full body illusions. These are the role of visual perspective and the role of visuotactile stimulation. The role of visual perspective is a key subject on which two major classes of results are diverging. Reports about the feeling of self-identification that occurs during experimentally induced OBEs suggest that the sense of ownership can be experienced over an artificial body seen from a third person perspective (3PP) (Lenggenhager et al., 2007, 2009; Aspell et al., 2009; Ionta et al., 2011). However, studies focusing on the full body ownership illusion report that a 3PP over the artificial body does not result in the illusion even when synchronous visuotactile stimulation is delivered (Petkova and Ehrsson, 2008; Slater et al., 2010; Petkova et al., 2011). The difference between these findings could be ascribed to differences in the experimental designs adopted or alternatively to the fact that different kinds of perceptual illusions are involved. The full body ownership illusion experienced in 1PP is described as the feeling of *owning* an artificial body, which substitutes the real body as the origin of perceptual sensations. (e.g., Petkova and Ehrsson, 2008; Slater et al., 2010). Experimentally induced OBEs have been characterized in different ways according to the specific experimental protocol adopted. Depending on the stroking mode, participants have one of two different experiences: (1) the visual 1PP and tactile sensations coincide in the same body—since there is the sensation of being embodied behind the location of the real body that is seen in front (chest stroking); (2) the visual 1PP and the origin of tactile sensations are divorced, since the visual 1PP is at the real body location, but tactile sensations are attributed to the fake body that is seen in front (back stroking). For a comparative study see Lenggenhager et al. (2009). The latter illusion has been shown to correlate with a recoding of self-location in space as measured by walking responses (Lenggenhager et al., 2007) and mental imagery (Lenggenhager et al., 2009), as well as with a remapping of the spatial representation of visuotactile stimuli as measured by the crossmodal congruency effect (CCE; Aspell et al., 2009). It has been further found to correspond with the specific activation of a number of brain areas such as the temporo-parietal junction (TPJ), the bilateral premotor cortex (PMC) and the medial sensorimotor cortex (Ionta et al., 2011; Lenggenhager et al., 2011). Despite this, the possibility that the sense of ownership and touch could be experienced over a body located in the far extrapersonal space is still under discussion. It has been proposed that self-identification during OBEs is not an actual perceptual illusion, but rather a form of self-recognition similar to the self-recognition of oneself in a mirror (Petkova et al., 2011; Ehrsson, 2012). Whether the controversy concerning the role played by visual perspective is grounded in the very nature of the illusion or in uncertainties associated with the experimental set-ups adopted remains to be clearly established.

The second main issue under debate concerns the role of multisensory stimulation as a trigger to elicit the illusion. A conclusion drawn in most studies is that synchronous visuotactile stimulation is necessary to elicit the illusion. This is the case for all the OBE's studies mentioned above. Petkova and Ehrsson (2008) also reported that, together with a 1PP over a humanoid artificial body, synchronous visuotactile stimulation is necessary to trigger the full body ownership illusion (see also, Petkova et al., 2011). Using an immersive virtual reality (IVR) set-up, Slater et al. (2010) came instead to a different conclusion. In their experiment participants wore a tracked HMD, receiving visual sensorimotor contingencies, meaning that the displayed field of view is continuously updated according to head position and orientation. Additionally, a virtual mirror provided synchronous visual feedback of head movements (visuomotor correlations). Participants received visuotactile stimulation either synchronously or asynchronously, and in both cases they experienced a strong full body ownership illusion. The fact that participants in this experiment could experience the illusion even when receiving asynchronous visuotactile stimulation seems to contradict the results of Petkova and Ehrsson (2008). However, there are two main differences between the two experimental set-ups. First, visual sensorimotor contingencies were provided in the IVR experiment, while in Petkova et al.'s experiment participants were exposed to a static field of view and were asked not to move their head, which was fixed looking down toward their body. Second, the virtual body in the IVR experiment had a realistic human body appearance, in term of skin texture and clothes, while in the other case the artificial body was a plastic mannequin. Whether these differences could explain the different conclusions drawn from the two experiments regarding the necessity or otherwise of visuotactile stimulation, is still not clear.

In this paper we report a set of experiments that systematically varies factors thought to contribute to the full body ownership illusion. Specifically, we consider the effect of visuotactile correlations and visual (head-based) sensorimotor contingencies, visual perspective and visual appearance. Our main aim was to fit previous results into a common framework and to propose a model that would allow assessment of the relative contributions and reciprocal interactions of such bodily signals to the full body ownership illusion.

## 2. Materials and methods

### 2.1. Overall design

Our study consisted of three experiments designed with the aim of exploring and disentangling the relative role of those factors that have been reported to be the building blocks of the full-body illusion: visual perspective, human-like bodily appearance, and visuotactile stimulation. Additionally, we consider the effects visual sensorimotor contingencies provided by head tracking, where the visual field displayed in the HMD is updated according to the tracked movements of the participant's head, in the same way as it would be in physical reality.

In experiment 1 we examined the effects of visuotactile and visual sensorimotor stimulation and their possible interaction. For this experiment we used a between-groups 2 × 2 design with two binary factors: visuotactile stimuli could be *synchronous* (VT) or *asynchronous* (⊥VT); head tracking could be *enabled* (HT) providing visual sensorimotor contingencies, or *disabled* (¬HT), which is with no head movements and with a fixed field of view, as in the set-up of Petkova and Ehrsson (2008). The design of this first experiment was mainly driven by the attempt to account for the difference in the results reported in Petkova and Ehrsson (2008) and Slater et al. (2010), since one important difference between the two studies concerns visual sensorimotor contingencies that are provided in the latter, but not in the former.

Experiment 2 was designed to explicitly test the hypothesis that visual perspective is a critical factor for the full body ownership illusion. For this we adopted a single-factor design, perspective, which had two levels (1PP vs. 3PP). Head tracking was set to HT and visuotactile to VT. This was to examine the effect of perspective in the otherwise most favorable condition for the illusion, i.e., with synchronous visuotactile stimulation and visual sensorimotor contingencies. If 3PP prevents the illusion in this case, we can safely extrapolate that the same will happen for other configurations where at least one of the two stimulations is in an incongruent mode (e.g., ⊥VT), or is not provided (e.g., ¬HT).

In experiment 3 we examine the impact of the level of realism for the appearance of the artificial body, comparing the effect of seeing a realistic human avatar (HA) vs. a plastic mannequin avatar (MA). The hypothesis was that the level of realism of the visualized body (in terms of skin texture and clothing) can modulate the intensity of the full body ownership illusion. As in the case of experiment 1, the design of this experiment has been motivated by the second critical difference among the experimental set-ups of Petkova and Ehrsson (2008) and Slater et al. (2010): the visual appearance of the fake bodies. We employed a single-factor design where the bodily appearance varied on two levels (HA vs. MA) with the other factors in the [1PP, ⊥VT, ¬HT] configuration. This choice enabled us to disentangle the hypothesized modulation of the illusion intensity by the visual appearance of the artificial body from the effects of multisensory and sensorimotor contingencies.

### 2.2. Implementation

The factors that were manipulated in the three experiments are summarized in Table 1: visual perspective (1PP vs. 3PP), bodily appearance (HA vs. MA), visuotactile stimulation (VT vs. ⊥VT), and visual sensorimotor stimulation from head tracking (HT vs. ¬HT). Although all the four factors vary on two levels, the three experiments planned for this study involve in total six conditions, as some factor combinations could be used for more than one experiment.

**Table 1:**
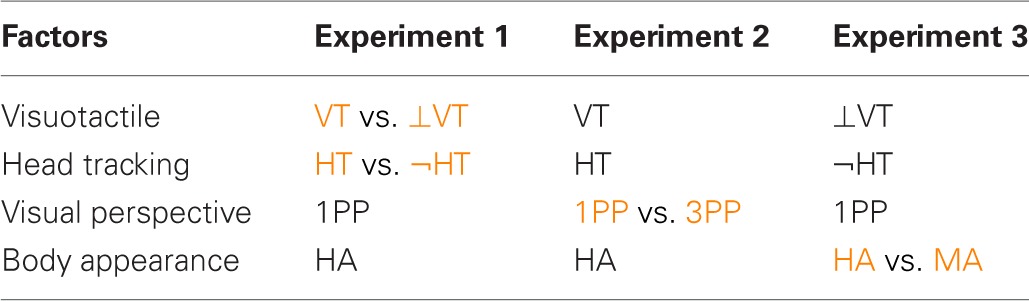
**Combinations of experimental factors adopted for the three experiments**.

*All experimental factors vary on two levels: “visuotactile” could be synchronous or asynchronous (VT vs. ⊥VT); “headtracking” could be enabled or disabled, providing or not visual sensorymotor contingencies (HT vs. ¬HT); “visual perspective” could be first person or third person perspective (1PP vs. 3PP); and “body appearance” could be either human or mannequin appearance (HA vs. MA). The variables in orange show the levels of the experimental factors, while the ones in black show the fixed configuration adopted for each experiment. There were nine participants in each of the four factorial cells of experiment 1, another nine participants in the 3PP condition of experiment 2, and another nine in the MA condition of experiment 3, resulting in a total of 54 participants*.

When head tracking was enabled, participants could explore the virtual environment and, looking down, they could see the virtual body when in the 1PP condition. In the ¬HT conditions the virtual scene was instead static and participants were instructed not to move their head and gaze direction, which was fixed looking down toward the virtual body.

**Figure 1:**
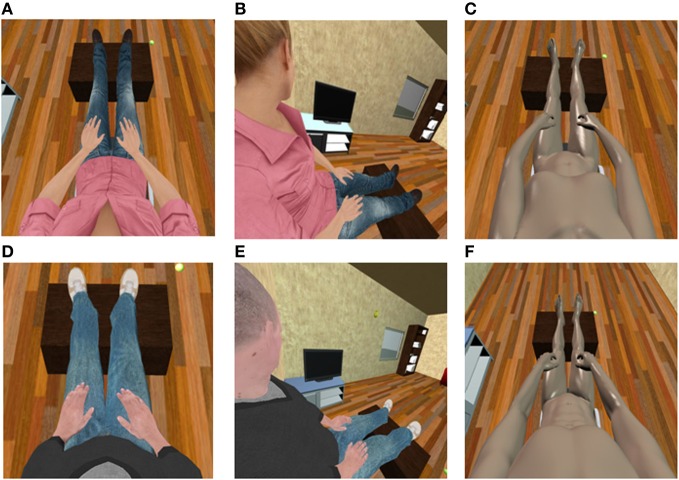
**Perspective levels and body appearances levels used for the different conditions of the experiments.** Gender-matched avatars were assigned to each participant. **[A,B (D,E)]** Show the two levels of the factor “perspective” for a female (male) participant: in the 1PP conditions **(A,D,C,F)** when looking down participants could see a virtual body co-located with their own physical body, while in the 3PP conditions **(B,E)** participants saw a virtual body in their near extrapersonal space when looking to the left. The two levels of the factor “bodily appearance” are shown in **[A,C (D,F)]** for a female (male) participant: in the HA conditions participants had a co-located virtual body with a realistic human appearance **(A,D)**, while in the MA condition a co-located virtual body resembling a plastic mannequin **(C,F)**.

Figures 1A,B (1D,E) show the perspective levels for a female (male) participant. For the case of 3PP, the point of view was shifted horizontally 40 cm away to the right; head tracking was enabled, so that participants could see the virtual body to their left, in their near peripersonal space (i.e., within reach). Participants in this condition also saw the avatar's head moving as their own while exploring the environment, which provided them with additional visuomotor correlations. Note that since the avatar's head followed the movements of the participant's head, participants never got to see the avatar's face (see Figures 1B,E). The avatars used to test the role of the body visual appearance are shown in Figures 1A,C (1D,F) for the case of a female (male) participant.

**Figure 2:**
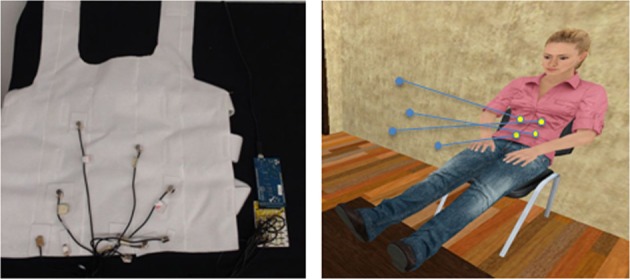
**The vibrotactile device used in the experiments.** The **left panel** shows the haptic vest used to deliver touch sensations: vibrators were located on the vest, mapping the location of the contact points of the ball's pre-recorded path with the avatar's chest. The **right panel** shows the paths followed by the virtual ball (blue lines) and its contact points with the avatar chest (yellow dots). In synchronous conditions the vibrators array was synchronized with the pre-recorded path, so that each vibrator was activated when the ball reached the corresponding path-chest contact point.

The apparatus used to implement the visuotactile stimulation is shown in Figure 2. Touch was delivered via mechanical vibrators attached to a haptic vest, while the seen touch was provided in the form of a ball bouncing on the virtual body along a pre-recorded path. The location of the vibrators on the haptic vest was adjusted for each participant, so as to match the contact points of the ball's pre-recorded trajectory with the chest of the avatar. In the VT condition each vibrator was activated for a short time interval (20 ms) when the simulator detected a collision of the ball with the avatar mesh in the corresponding point (Spanlang et al., 2010; Pomes et al., 2012). The time delay of the vibrator activation with respect to the virtual collision corresponds to the communication time between the machine running the IVR software and the haptic vest and was negligible (<10 ms). The asynchronous condition was implemented by randomly activating the vibrators when the virtual tapping was visualized, so that the touch and the visual collisions were not correlated.

### 2.3. Participants

A total of 54 naïve subjects were recruited for the study, 9 for each of the six conditions over the three experiments (Table 1). Participants were recruited from the University campus or from a database of persons who had previously agreed to being contacted for participation in VR experiments. All subjects participated in only one experiment—i.e., it was a between-groups design. Experiment 1 tested 36 subjects (18 female, mean age 23.3, SD 5.1). Experiments 2 and 3 each involved 18 subjects (experiment 2: 7 female, mean age 29.4, SD 7.6; experiment 3: 8 female, mean age 25.4, SD 4.6). Note that the [VT, HT] group in experiment 1 has the same experimental configuration as the 1PP experimental group in experiment 2. For this reason we could use the same experimental group independently for the two experiments. The same applies for the [⊥VT, ¬HT] group in experiment 1 and the HA group in experiment 3.

The study was performed according to institutional ethics and national standards for the protection of human participants, and it was approved by the Comit'e 'Etico de Investigaci'on of the University of Barcelona. All participants signed an informed consent form and filled in a pre-questionnaire to collect demographic information before taking part in the experiment. They were then assigned arbitrarily to one of the six conditions and were paid €10 after the experiment was concluded. In the pre-questionnaire all participants were asked “Have you ever experience ‘virtual reality’ before?”, with possible responses on a scale from 1 (no experience) to 7 (extensive experience). The response was on average low (median value 1), with only 6 participants out of 54 scoring 4 or higher values.

### 2.4. Experimental procedure

Participants were instructed to sit comfortably on a chair with their legs resting on a footstool. They entered the virtual environment using a wide field-of-view head-tracked, HMD. We used a NVIS nVisor SX111 HMD with dual SXGA displays of 76°H × 64°V degrees field of view per eye, corresponding to a global field-of-view of 111° horizontal and 60° vertical, with a resolution of 1280×1024 per eye displayed at 60 Hz. Head tracking was performed by a 6-DOF Intersense IS-900 device. Depending on the gender and the condition assigned, each participant was exposed to one of the scenes displayed in stereo within the HMD, shown in Figure 1. Participants in every condition were instructed to not move their body from the neck down for the whole duration of the experiment. Participants in HT conditions were explicitly asked to turn their head and look around the environment, while those in ¬HT conditions were asked not to move the head and not to gaze around.

**Figure 3:**
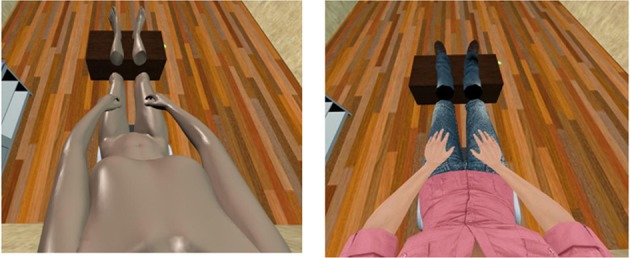
**Snapshots of the legs separation event are shown for the mannequin and human appearance modes in the case of a female participants.** The legs separation event lasted 10 s during which the legs moved away from the body reaching a maximum distance of 1 m. The legs returned to their original position immediately after the 10 s, recomposing a whole body.

After an adaptation period of about 30 s, a yellow ball appeared in the scene and moved toward the virtual body. When it struck the body it activated the vibrators on the haptic vest (in synchronous or asynchronous mode according to the condition). Then it bounced off the body moving away again. This tapping phase lasted for an interval of 2 min. In this phase participants were instructed to pay attention to the virtual body and to the tapping ball. At the end of the tapping phase the ball disappeared and the vibrators stopped. Participants were then asked to focus their attention on the virtual body, and after about 30 s they were exposed to a disturbing event: the lower legs slowly moved away from the rest of the body for 10 s, covering a distance of about 1 m in the virtual room, and after that they returned to the original position to recompose the whole body (see Figure 3). The choice of this particular kind of disturbing event instead of a more classic threat to the virtual body is based on two main motivations: first, we wanted to avoid any kind of threat (even if virtual) directed to the spatial location of the real body; second, we looked for an event that explicitly breaks the integrity of the whole body.

### 2.5. Response variables

#### 2.5.1. Questionnaire

For all the three experiments we used a 7-item questionnaire designed to assess the level and quality of the illusion experienced by the participants. Each question was scored by participants on a given scale from 0 (not at all) to 7 (very much). The choice of an even number of points on the Likert scale was meant to force scoring either on the agreement or on the disagreement side. Having four points on each side further allowed for various degrees of agreement/disagreement, so as to detect differences in the intensity of the experienced sensations (Cohen et al., 2011).

The questionnaire is shown in Table 2. The items were formulated by adapting questions from previous experiments on full body illusions (Lenggenhager et al., 2007; Petkova and Ehrsson, 2008; Slater et al., 2010). The main critical item is the one tagged as *mybody*, which relates directly to the feeling of owning the virtual body. The *clothing* item was also meant to test the feeling of ownership following Slater et al. (2010) where it was found that the body ownership illusion was highly correlated with the illusion of wearing the same clothes as the virtual character. The items *stress* and *discomfort* were formulated to detect the subjective response to the disturbing event. These two apparently similar statements were meant to distinguish the feeling of weirdness (something weird is happening to my body, but it is not stressful) from the feeling of stress that is a state of anxiety. The *catch* item tested the desire of participants to move and act in the virtual environment. The *touch* item was meant to test the appropriate implementation of the visuotactile stimulation: the proper functioning the vibrotactile vest and its integration in the virtual environment had to be demonstrated by having low/high scores to the *touch* item in the asynchronous/synchronous modalities. Finally, the *twobodies* item was a control question.

**Table 2:** **Questionnaire**.

**Variable name**	**Item**	**Statement category**
*Mybody*	I felt that the body I saw was my body	Ownership
*Clothing*	I felt that I was wearing different clothing to my real clothing	Ownership
*Stress*	I was stressed when I saw the legs coming apart	Psychophysical reaction
*Discomfort*	I felt physical discomfort when the legs were coming apart	Psychophysical reaction
*Catch*	I wanted to try to catch the yellow ball	Interaction in VR
*Touch*	It seemed as though the touch I felt was caused by the yellow ball touching my body	Implementation
*Twobodies*	I felt that I had two bodies	Control

#### 2.5.2. Heart rate deceleration

We monitored the physiological behavior of the participants by recording their electrocardiogram (ECG) throughout the whole duration of the experiment. This was used to obtain a measure for the heart rate deceleration (HRD) in various stages of the experiment. HRD has been previously shown to be a response variable that significantly correlates with states of stress and anxiety induced by sudden unpleasant stimuli (Bradley et al., 2001; Cacioppo et al., 2007), and has been successfully used in previous studies as a physiological correlate of the full body ownership illusion (Slater et al., 2010) and other perceptual illusions (e.g., Tajadura-Jiménez et al., 2012). In our study, instead of using a sudden threat, we have adopted a threat to the integrity of the body that is extended in time, i.e., the legs separating from the rest of the virtual body, in slow motion, over a period of 10 s. For this reason it is not possible to identify the precise moment at which participants noticed this event. To account for this, we introduced a new procedural definition for the HRD.

The ECG signal was sampled at 256 Hz using the g.tec portable bio-signal acquisition device g.MOBIlab+^1^. ECG signals were processed to extract the heart rate (HR) by first applying the automatic search for QSR complex implemented in the gtec biosignal analysis software g.BSanalyze^2^, and then by visually inspecting the search results to correct for possible missing or false identifications. The HRD response to the legs separation, HRD_legs_, was calculated within the period of 6 s immediately after the legs started to move away from the rest of the virtual body, by looking for the first sustained deceleration event occurring in this time window (i.e., the earliest time interval in which the HR signal was monotonically decreasing). In order to have a reliable detection of sustained deceleration associated to the unpleasant event, we imposed two constraints: (1) the extent of the time interval of monotonic deceleration had to be larger than 1.5 s, so as to ensure that this was a sustained deceleration; (2) the starting point of the sustained deceleration should be at most 2 s away from the starting edge of the 6 s period. The latter constraint was meant to account for the possibility that the participants did not immediately detect the onset of the unpleasant event. Once we identified the deceleration event in the HR signal, we calculated the HRD as: Δ HR/Δ*t*, with Δ HR = HR(*t*_max_) − HR(*t*_min_) and Δ*t* = *t*_min_ − *t*_max_. Here *t*_min_ and *t*_max_ denote the location in time of the minimum and maximum, respectively. For those cases in which the HR signal was monotonically increasing within the whole 6 s window, we have used an analogous procedure to obtain negative HRD values, describing in fact a heart rate acceleration.

As the HRD measurements are expected to be subject-dependent, we have further estimated a measure for the HRD baseline of each participant. In order to obtain a representative baseline value, we have applied the same procedure described above for measuring HRD from single sustained deceleration event, in 10 different time windows (each of 6 s) within the 20 s rest period preceding the legs separation. The baseline value, HRD_base_ was defined as the mean of these 10 HRD measures.

## 3. Results

### 3.1. Statistical methods

Since the responses to the questionnaires are ordinal and not interval scaled, we used non-parametric tests for the questionnaire analysis. Data from experiment 1 were analyzed with the Friedman test for non-parametric analysis of 2 × 2 designs for testing the effect of each factor, while taking into account the other as a blocking factor. Note that the Friedman test does not allow detection of interaction effects. When comparing two samples, as in experiments 2 and 3 and in some post analysis of experiment 1, we used the Mann–Whitney U test.

The HRD data were inspected in order to look for changes induced by the legs separation event, one that obviously affected the integrity of the body, with respect to the baseline. We performed analysis of variance (ANOVA) comparing the change in HRD induced by the threat and measured as Δ HRD = HRD_legs_ − HRD_base_, between the experimental conditions of each experiment. Finally, we bring various results together in one simple path analysis model that helps understand the results of the experiments.

All analysis was carried out using the Stata 12 statistical package^3^.

### 3.2. Experiment 1: multimodal contingencies

#### 3.2.1. Questionnaire

**Figure 4:**
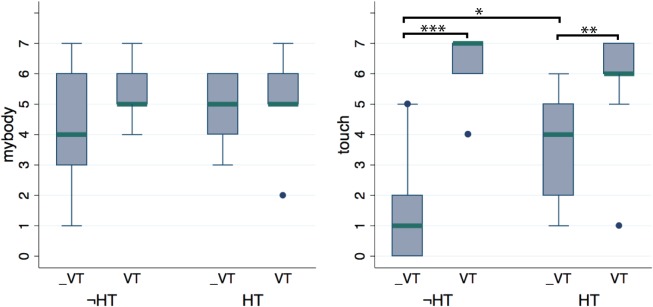
**Questionnaire data from experiment 1, in which we investigate the role of viusotactile and visual sensorimotor contingencies.** No difference was found in the *mybody* item showing that the illusion was not affected by either of the two multisensory modalities (when a 1PP is provided over a realistic virtual body). Significant differences were found for the *touch* item with respect to the visuotactile mode, for both levels of the head tracking factor. This showed that the vibrotactile device used to deliver touch sensations was effective. ^*^p < 0.05, ^**^p < 0.01, ^***^p < 0.001 from Mann–Whitney test.

Experiment 1 was a 2 × 2 design with factors visuotactile (VT vs. ⊥VT) and head tracking (HT vs. ¬HT). Figure 4 shows the boxplots comparing the distributions of scores in the four conditions for relevant items. It is evident that there is no significant difference between the four conditions with respect to the sensation of body ownership (item *mybody*). The Friedman test applied to the *mybody* item confirmed that there is no significant difference with respect to both the visuotactile and head tracking factors. Median scores were at least 4 in all the four groups, suggesting that participants experienced some illusion of body ownership over the virtual body whatever the visuotactile and head tracking modes. Responses to the *clothing* item also received high scores in all the four conditions (all medians at least 4).

The *stress* and *discomfort* items had median values below 4 and no significant difference was found between the four conditions. Nevertheless, verbal reports revealed that most participants experienced a strange sensation while seeing the virtual legs separating, a fact often corroborated by spontaneous exclamations and verbalizations during and after the legs separation.

Scores to the *touch* item were significantly higher for the VT conditions than for the ⊥VT, for both the HT (*p* = 0.009, Mann–Whitney; average ranks: 6.28 for ⊥VT, 12.72 for VT) and ¬HT (*p* = 0.001, Mann–Whitney; average ranks: 49 for ⊥VT, 122 for VT) groups, showing that our implementation of the visuotactile stimulation via automatic mechanical vibrators was effective. On the other hand participants had significantly greater scores in the HT compared to the ¬HT mode, in the ⊥VT mode (*p* = 0.048, Mann–Whitney; average ranks: 7.06 for ¬HT, 11.94 for HT), while no significant difference between the HT levels is present in the VT mode group. The Friedman test applied to the *touch* item detected significant differences with respect to both the VT factor (*p* < 0.0005) and HT factor (*p* = 0.042). It is noticeable that the asynchronous visuotactile stimulation was clearly perceived as wrong (median score is 1) only in the [⊥VT, ¬HT] condition, while in the [⊥VT, HT] condition the same asynchronous stimulation till had a median score of 4. Hence some participants still perceived this as a touch sensation congruent with the collision of the virtual ball with the body—as indicated from their reports and scores that reached as high as 6.

Scores to the *catch* item were significantly greater in the HT condition than in the ¬HT for the ⊥VT group (*p* = 0.014, Mann–Whitney; average ranks: 6.5 for ¬HT, 12.5 for HT). No significant difference was found for the VT group. Analogously, a significant difference with respect to the visuotactile factor was found for the ¬HT group (*p* = 0.015, Mann–Whitney; average ranks: 6.5 for ⊥VT, 12.5 for VT) but not for the HT one. The Friedman test detected significant differences between the scores to the *catch* item with respect to the head tracking factor (0.005), but not for the visuotactile one. It is interesting to note that the median score was much smaller only in the [⊥VT, ¬HT] condition. For the other three groups, when congruent multisensory and/or visual sensorimotor correlations were provided (VT or HT or both), the scores tended to be quite high with medians at least 4. This showed that the desire to move and act in the virtual environment was mainly driven by multisensory/sensorimotor correlations and did not depend on the sense of ownership over the virtual body alone.

Since the level of ownership turned out to be independent of two experimental factors, we can further examine whether there are some undetected differences in the scoring to the *mybody* item by combining samples. We first compared the two visuotactile levels irrespectively of the head tracking mode and vice versa (18 subjects per group). In both cases the Mann–Whitney test found no significant difference. The same procedure was applied to the other questionnaire items for which no dependence on either VT and HT was found (all but *touch* and *catch*). This confirmed previous findings, i.e., that the feeling of ownership over the virtual body did not depend on either the visuotactile and the head tracking modes signals.

#### 3.2.2. Heart rate deceleration

Two-Way ANOVA of ΔHRD showed that neither of the two factors, nor their interaction, was significant in modulating the HRD response when threatening the integrity of the body [VT: *F*_(1, 32)_ = 0.40, *p* = 0.53; HT: *F*_(1, 32)_ = 1.32, *p* = 0.26; interaction: *F*_(1, 32)_ = 1.80, *p* = 0.19]. We have further checked specifically for the effect of the visuotactile factor, collapsing together the two headtracking levels. The same was done for testing further the effect of the headtracking factor, grouping together the visuotactile levels. In both cases no significant differences were found [VT: *F*_(1, 34)_ = 0.39, *p* = 0.54; HT: *F*_(1, 34)_ = 1.31, *p* = 0.26].

### 3.3. Experiment 2: perspective

#### 3.3.1. Questionnaire

**Figure 5:**
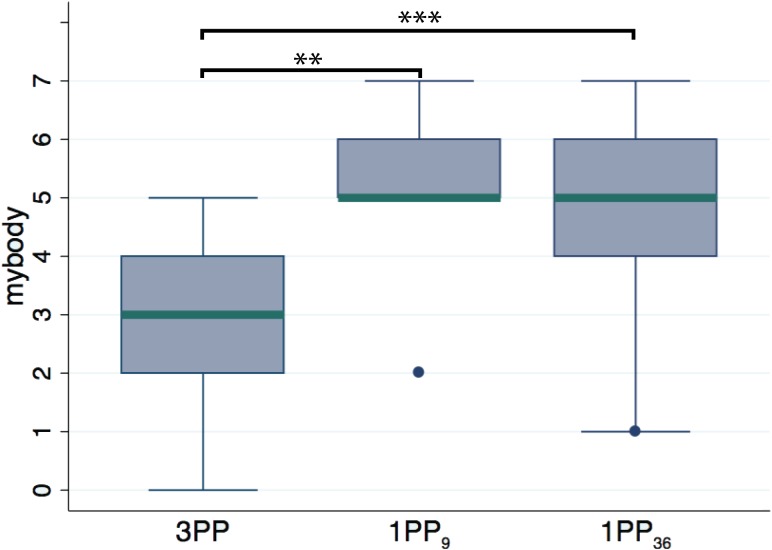
**Questionnaire data from experiment 2, in which we compare groups with different perspective levels.** 3PP (9 subjects), 1PP_**9**_ (9 subjects) and 1PP_**36**_ (36 subjects, where all 1PP conditions were grouped together irrespectively of the visuotactile and head tracking modes). The significant difference in the *mybody* item showed that 1PP is an essential condition for the full body ownership illusion. ^**^p < 0.01, ^***^p < 0.001 from Mann–Whitney test.

Figure 5 shows the boxplot comparing scores to the *mybody* items from 1PP and the 3PP groups. From the figure one can see a noticeable effect of visual perspective. Comparing the two groups, [1PP, VT, HT] vs. [3PP, VT, HT] with 9 participants each, a Mann–Whitney test returned a significant difference (*p* = 0.004; average ranks: 5.89 for 3PP, 13.11 for 1PP). For all the other items no significant differences were found.

Given the results from experiment 1, i.e., visuotactile and head tracking modes have no effect on the ownership illusion, we combined the groups across these levels in order to increase the power of the test. The Mann–Whitney test comparing the 3PP group (9 subjects) with that of 1PP with both visuotactile and head tracking levels merged (36 subjects), resulted in significant differences in the perspective levels for both the item *mybody*, now with greater significance (*p* = 0.0007; average ranks: 10 for 3PP, 26.25 for 1PP), and *clothing* (*p* = 0.024; average ranks: 14.28 for 3PP, 25.18 for 1PP).

#### 3.3.2. Heart rate deceleration

One-Way ANOVA of Δ HRD resulted in no significant effect of the perspective factor on the HRD response when threatening the integrity of the body [*F*_(1, 16)_ = 1.21, *p* = 0.26]. No difference was found when further comparing the 3PP group with the 1PP_36_ group [*F*_(1, 16)_ = 0.10, *p* = 0.75].

### 3.4. Experiment 3: bodily appearance

#### 3.4.1. Questionnaire

**Figure 6:**
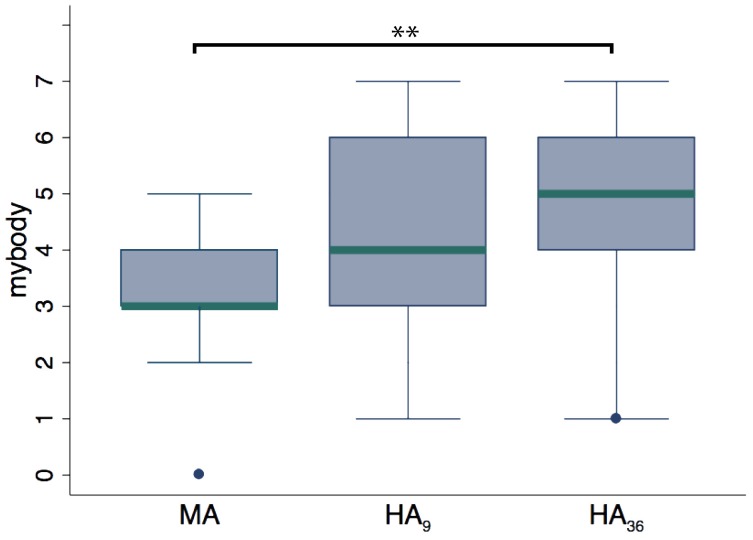
**Questionnaire data from experiment 3, in which we compared two groups that had different virtual bodies.** Mannequin appearance: MA (9 subjects); human appearance: HA_9_ (9 subjects) and HA_36_ (36 subjects, where all the human appearance conditions in the 1PP were grouped together irrespectively of the visuotactile and head tracking mode). Significant differences were found when comparing the MA group with the HA_36_ group. This showed that a realistic body appearance significantly enhances the illusion experience. ^**^p < 0.01 from Mann–Whitney test.

Figure 6 shows the boxplots comparing the scores to the item *mybody* for the HA and MA groups. No significant differences were found between the two groups in the [1PP, ⊥VT, ¬HT] configuration (9 subjects per group; HA_9_ vs. MA). However, since there was no dependence of the ownership illusion on the visuotactile and head tracking factors (results from experiment 1), we could group the levels of these two factors. Doing so we obtained a larger sample of 36 subjects for the HA (HA_36_) condition to be compared with the MA group (9 subjects). In this case we found significant difference for both the items *mybody* (*p* = 0.003; average ranks: 11.44 for HA, 25.89 for HA) and *clothing* (*p* = 0.011; average ranks: 13.17 for HA, 25.46 for HA). This suggests that a realistic body appearance significantly enhances the ownership illusion. It is important here to note that despite the differences found in the two visual appearance groups and despite the asynchronous visuotactile stimulation, the sense of ownership was present to some extent even when the virtual body had a mannequin appearance, as shown from the fact that participants in the MA group had a median score of 3 on the *mybody* item.

#### 3.4.2. Heart rate deceleration

One-Way ANOVA showed no significant difference in Δ HRD between the two body appearance groups [*F*_(1, 16)_ = 1.24, *p* = 28]. No difference was found when further comparing the MA group with the HA_36_ group [*F*_(1, 16)_ = 0.60, *p* = 0.44].

### 3.5. Path analysis

**Figure 7:**
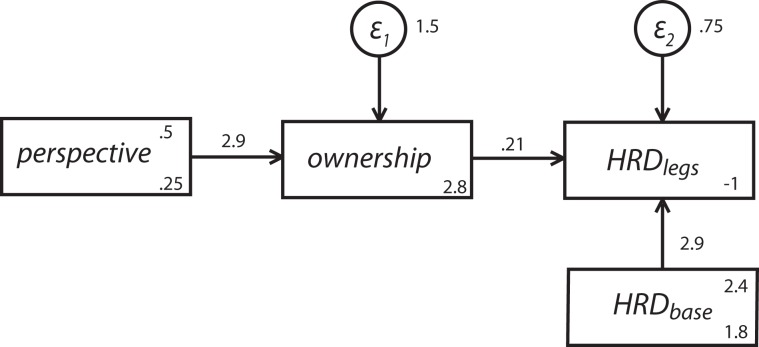
**Path analysis model that fits data from experiment 2.** The boxes represent the variables: perspective can be 0 or 1 for 3PP and 1PP, respectively; ownership is the response to the questionnaire item *mybody*; HRD_base_ is the heart rate deceleration baseline, and HRD_legs_ is the heart rate deceleration measured right after the legs start separating. The circles represent random error terms.

It is not too surprising that we found no apparent effect on the HRD of the legs separation threat to the body, since there was no differential effect on body ownership of most of the factor levels. We would expect that only in conditions of a high body ownership illusion would the threat to the integrity of the body cause the type of abhorrent response that might be reflected in HRD_legs_. We found a strong impact on ownership only for the manipulation that involved perspective (experiment 2). Hence in this case we would expect an impact on HRD_legs_. However, the situation is not straightforward since the HRD during the event would be likely to be associated with the baseline HRD but also and separately be associated with the level of ownership, which itself is impacted by the manipulation in perspective (1PP or 3PP). For this reason we used path analysis to further explore the possibility that HRD_legs_ is indirectly affected by perspective through the feeling of ownership, a possibility that is not testable using simple analysis of variance. We propose the path model in Figure 7, in which perspective directly affects ownership, ownership affects HRD_legs_, and HRD_legs_ further depends on HRD_base_. The latter path takes into account intrinsic HRD differences between individuals. We used the scores from the questionnaire item *mybody* as a quantitative estimate of ownership. Note that here we treat *mybody* as an interval scaled variable; even if this is not formally justified, it is an approximation usually adopted as it provides useful exploratory tool.

We used the structural equation modeling software of Stata 12 to estimate the coefficients and the corresponding significant levels, using the asymptotic distribution free option, since we had no reason to suppose that the variables involved follow a multivariate normal distribution.

The model proposed provides a very good fit to the data. A test of the goodness of fit of the model against the saturated model showed a good fit, with χ^2^(2) = 2.04, *p* > 0.36. All estimates are summarized in Table 3. This result shows how the feeling of ownership is strongly affected by perspective as already discussed. Additionally it shows that, even though there is there is not a direct significant impact of perspective on HRD, the response to the threat in terms of HRD increases with ownership. Also, as expected, the HRD response to the threat is highly correlated with HRD_base_, reflecting the intersubject HRD variability.

**Table 3:** **Coefficients, standard errors, and *p*-values estimates from path analysis applied to the model in Figure 6, using data from experiment 2**.

**Variable**	**Coefficients**	**Standard errors**	***P***
**OWNERSHIP**			
Perspective	2.89	0.60	<0.001
Intercept	2.79	0.51	<0.001
**HRD_legs_**			
Ownership	0.21	0.70	0.003
HRD_base_	0.82	0.60	<0.001
Intercept	−1.03	0.27	<0.001

The factor perspective then has an effect on HRD mediated through the subjective level of ownership as measured by the questionnaire variable *mybody*.

## 4. Discussion

Our study contributes several key findings regarding the full body ownership illusion. Three experiments were specifically designed to pinpoint the main perceptual cues that contribute to the full body ownership illusion, to determine their specific role and assess their interaction. Motivated by previous results in the literature, we focused on the following main perceptual cues: visuotactile and visual (head-based) sensorimotor stimulation, visual perspective, and body appearance. The main findings are compatible with the following statements:

- First person perspective is a necessary condition for the full body ownership illusion.
- The full body ownership illusion can result from the sole effect of seeing a realistic virtual body in the same location and posture as the physical body, a configuration that contributes toward the experience of correct visuoproprioceptive cues, with no need for the additional contribution of congruent multisensory and/or head-based sensorimotor cues.
- The appearance of the virtual body and more specifically its level of realism in terms of skin texture and clothes, can influence the strength of the full body ownership illusion.
- Multisensory and/or sensorimotor contingencies can influence the level of the full body ownership illusion, having a reinforcing effect when congruent and a damping effect when incongruent.
- The full body ownership illusion can modulate the way touch is perceived, in that asynchronous visuotactile cues can be consciously perceived as correct, especially when there is first person perspective and a realistic virtual body.

Note that we did not directly explore the effect of visuomotor correlations, as participants could only move the head and were not allow to move the rest of the body. We speculate that congruent visuomotor correlations provide powerful multimodal cues that have a similar—though stronger—effect on the ownership illusion as the multisensory and head-based visual sensorymotor contingencies explored in our experiments. This speculation needs to be further tested in a future study.

In the following we discuss the above findings in the context of the relevant literature, highlighting the new findings with respect to previous studies.

### 4.1. The role of perspective

In experiment 2 we found clear evidence for 1PP being a critical factor for eliciting the full body ownership illusion. The questionnaire results showed a strong significant difference between participants with 1PP and 3PP, in that only the first group experienced the illusion of ownership with respect to the virtual body. HRD data further support this finding (see section 3.5). Moreover, our experimental set-up showed that 3PP inhibited the illusion even when various form of congruent multisensory and sensorimotor cues were provided. Therefore, as a general result, it could be said that first person perspective over the fake body is a necessary condition for the onset of the full body ownership illusion. Our results bring also further insights on the issue raised in Petkova et al. (2011) as to whether 3PP prevents the illusion because it violates the 1PP as such, or less restrictively, because (as in their experiment) the body to be owned is seen in the far extrapersonal space. In our experimental set-up, the virtual body is seen from 3PP and it is located within the peripersonal space. The finding that the illusion is suppressed in this set-up demonstrates that violation of 1PP over the fake body is sufficient to prevent the full body ownership illusion, even if the body is seen in the peripersonal space.

Our results are consistent with previous findings from studies on both the RHI and the full body ownership illusion. In the RHI paradigm, experiments by different groups have investigated the effect of displacing the position of the artificial hand with respect to the real physical one in order to determine the role of spatial congruency between visual and proprioceptive information. For the sake of the present discussion, the majority of these manipulations could be interestingly reinterpreted in terms of perspective over the body-part to be integrated, having 3PP for those cases in which the fake limb position violates constraints from 1PP, i.e., when the limb is outside the peripersonal space (Armel and Ramachandran, 2003; Lloyd, 2007) or in anatomically impossible configurations, e.g., at 180° (Ehrsson et al., 2004) and when handendness does not match (Tsakiris and Haggard, 2005). By doing this, the result is that all those cases in which the fake hand position violates constraints from 1PP have been found to work against the illusion, supporting the general conclusion that first person perspective is indeed a critical factor for eliciting the illusion of ownership over an external object. Analogously, studies focused on the full body ownership illusion showed that the illusion could not be induced in various configurations of 3PP, e.g., seeing the body laying in extrapersonal space (Petkova et al., 2011) or seeing the body located to the side in peripersonal space (as in our experiment 2). In both configurations, participants did not experience the illusion despite synchronous visuotactile stimulation and/or visual sensorimotor contingencies being provided.

A second group of studies reported, however, different conclusions on the role of visual perspective in ownership illusions. Preston and Newport (2012) presented an experimental setting in which participants experienced an illusion over their own arm filmed from a distance of 2 m and displayed on a screen (the arm image in the video was also manipulated so to show it elongated). Similarly, a number of studies concern experimentally induced OBEs, in which participants experience self-identification with a virtual full-body seen from a 3PP (Lenggenhager et al., 2007, 2009; Aspell et al., 2009; Ionta et al., 2011). The resulting sense of ownership and touch experienced over a body located in far extrapersonal space is in contradiction with the results reviewed above.

How can these two classes of results be compatible with each other? A possible explanation that has been recently proposed (Petkova et al., 2011; Ehrsson, 2012) is that the self-identification experienced during OBEs is in fact a form of visual self-recognition rather than a somatic ownership illusion, similar to the experience of recognizing oneself in a mirror. An element in favor of this interpretation can be found in the seminal experiment from Lenggenhager et al. (2007), where the same stroking protocol was applied while visualizing either the filmed participant's body or that of a plastic mannequin. Although in both cases a significant difference was found among the synchronous and asynchronous stroking modes, the actual scores of the questionnaire item related to self-identification were lower in the mannequin condition (Lenggenhager et al., 2007—supporting online material). This seems to suggest that the self-identification actually occurred only for participant seeing their own filmed body, supporting the “self-recognition” hypothesis. On the other hand, there are several objective measurements that provide evidence for the back-stroking induced OBE to be an authentic somatic illusion. Participants experiencing this illusion seem to undergo a remapping of the visuotactile receptive field (RF) (Aspell et al., 2009) and of the self-location in an allocentric spatial reference frame (Lenggenhager et al., 2007, 2009). A selective activation of the TPJ analogous to that occurring in neurological OBEs (Ionta et al., 2011), and of bilateral PMC and medial sensorimotor cortex (Lenggenhager et al., 2011) have also been found. We suggest here that the full body ownership and the back-stroking OBE are in fact two different kinds of body illusions, which involve the activation of different neural patterns (see section 4.4). A rigorous comparison of the two illusions requires, however, a dedicated experimental set-up and is beyond the scope of this paper.

A recent study of patients with the somatoparaphrenic delusion of disowning their own left arm (Fotopoulou et al., 2011) provided further insights on the role of visual perspective. It showed that the disownership of the arm in these patients can be suddenly extinguished when the arm is seen from 3PP (in a mirror). Disownership was reinstated, however, a few seconds after switching again to a 1PP over the arm. These results seem to suggest that the sense of ownership requires the activation of a perceptual mechanism specific to 1PP that is impaired in somatoparaphrenic patients. When the perspective is switched to 3PP with the use of mirrors, the visual processing of the own body undergoes a spatial transformation, and it may no longer require the impaired mechanisms responsible for the perceived ownership/disownership. Given the prompt switching between the sense of ownership and disownership, when changing from 1PP to 3PP and vice versa, this mechanism is likely to be purely perceptual and bottom–up. We propose that a plausible candidate for this is provided by consistent visuoproprioceptice cues and by the resulting pattern of neural activation. We discuss this possibility in the following section as well as in section 4.4.

### 4.2. The role of multimodal contingencies

Results from experiment 1 showed that subjects in an immersive virtual environment may experience a sense of ownership over a highly realistic virtual body seen from a first person perspective, independently of the visuotactile and head-based visual sensorimotor cues received. This is supported from both questionnaire and HRD responses to the event disrupting the integrity of the whole virtual body; no significant differences between the four groups were found in either measurement. This suggests that, when the body is not moving, the effect of congruent visuoproprioceptive cues alone, as provided by having a high degree of spatial overlap between the physical body and the realistic virtual body, is a sufficient condition for inducing a full body ownership illusion. Since the ownership illusion occurs when there is congruent visuoproprioceptive feedback, for a static body, then it should be all the more powerful when there is additionally congruent visuomotor feedback—so that the virtual body moves synchronously with, and spatially matches, real body movements. Note that the requirement for a high degree of overlap between the physical and virtual bodies is a stronger constraint than 1PP by itself, so that it is possible to have 1PP and no congruent visuoproprioceptive cues at the same time. For example, one could have 1PP over a virtual body with a plausible body posture that is nevertheless different from the posture of the obscured physical body, or 1PP over a human-like body that have different size and/or body proportions with respect to the real body. While 1PP seems to be a necessary condition for experiencing an ownership illusion, other work seems to suggest that the exact position and posture of the real and virtual bodies (body parts) do not need to coincide in space (e.g., de la Peña et al., 2010; Petkova et al., 2011). Somatic illusions can be experienced in such configurations if additional congruent multisensory and/or sensorimotor cues are provided, and in these cases proprioception can be altered and shifted toward the virtual body, as shown by measurements of the proprioceptive drift in the RHI (e.g., Botvinick and Cohen, 1998) and OBEs (e.g., Lenggenhager et al., 2007), as well as reports of changes in the perceived body posture (de la Peña et al., 2010). With our experiment we have shown that when there is a high degree of overlap between the real and virtual body, the illusion can be experienced with no need for additional congruent multisensory and/or sensorimotor cues.

Our results further show that the illusion arising from having a high degree of spatial overlap between the virtual and physical bodies can be sustained, even when asynchronous visuotactile stimulation is delivered to the participants and in the absence of any other form of congruent multimodal stimulation. As a further interesting outcome, we found that having or not visual sensoriomotor contingencies from head tracking affected the way participants perceived touch; in the group that received visual sensorimotor contingencies from head tracking throughout the experiment, the asynchronous visuotactile stimulation was not perceived as completely wrong, while when head movements were not allowed and the field of view was static the same visuotactile stimulation was clearly reported to be wrong. This is an important finding that clearly indicates that there is an interaction effect in the way these multimodal stimulations are processed, and that the way touch is consciously perceived can be modulated by the processing of other perceptual cues, possibly via the onset of the illusion.

Altogether, our results present various novel insights with respect to previous studies. In fact, it has been extensively reported that the processing of congruent visuotactile and/or sensorimotor stimulation is a necessary condition for the experimental elicitation of bodily illusion in healthy subjects. This is the case for most of the reported experiences of extracorporeal object assimilation (Botvinick and Cohen, 1998; Schaefer et al., 2009; Guterstam et al., 2011) and of body deformation illusions (Lackner, 1988; Ehrsson et al., 2005; Schaefer et al., 2007; Newport and Preston, 2010; Normand et al., 2011; Kilteni et al., 2012; Preston and Newport, 2012), as well as of full body ownership and out-of-body illusions (Lenggenhager et al., 2007, 2009; Petkova and Ehrsson, 2008; Aspell et al., 2009; Ionta et al., 2011). In some cases, it has been noticed that incongruent multisensory information can be assimilated without destroying the illusion. For example, Slater et al. (2010) found that asynchronous visuotactile stimulation did not prevent a full body ownership illusion in an IVR setting where participants had 1PP over the virtual body and received visual sensorimotor congruent stimulation from head-tracking.

The first question that arises when comparing our results with those from previous studies concerns the reason why previous studies have repeatedly found that synchronous visuotactile (or head-based sensorimotor) stimulation was a necessary condition for eliciting a body ownership illusion, while we have found that this is not the case. There are two main points that may answer this question. First, the hypothesis that “the sole effect of congruent visuoproprioceptive cues, provided by a high degree of spatial overlap between the physical and virtual bodies, is a sufficient condition for inducing a full body ownership” has never been tested explicitly. The RHI paradigm intrinsically involved the use of visuotactile stimulation, with the control condition being the asynchronous mode rather than a “no touch” condition. This paradigm has been extended automatically to the case of full body illusions. In spite of this, it has been shown that the RHI can persist during period in which visuotactile stimulation was not delivered (Hohwy and Paton, 2010). The experimental design included nevertheless an initial phase of synchronous visuotactile stimulation that may have induced the illusion, which then persisted. Importantly, this experiment used stereo goggles that allowed a high degree of spatial matching between the real and virtual hands. In the same study, it was shown that after a period of synchronous visuotactile stimulation participants kept perceiving touch sensations when real touch was no longer delivered on the real hand, but appeared to be applied on the virtual hand. These findings are in perfect agreement with our results. Second, the reason for which other studies did not come to a similar conclusion can also be due to the fact that in most RHI experiments, apart from those using immersive stereo displays, the spatial locations of the real and artificial hand cannot perfectly coincide and consequently the visuoproprioceptive information provided is not fully congruent. It is interesting to note that all those experiments in which the ownership illusion was not disrupted by incongruent visuotactile stimuation (Hohwy and Paton, 2010; Slater et al., 2010; our experiment 1), used stereo vision, thus achieving a high degree of spatial overlap between the real and the virtual body (or body part) and, additionally, found that the asynchronous visuotactile information was not consciously perceived as wrong.

In order to have a unitary scenario that would allow the unification of the findings of different studies, we further need to address the following question: why other studies in which the physical and virtual bodies were highly spatially coincident (Petkova and Ehrsson, 2008; Petkova et al., 2011) found synchronous visuotactile stimulation to be necessary for the full body ownership illusion? To answer this question we move to the next section, where we discuss the role of the visual appearance of the virtual body.

### 4.3. The role of body appearance

We have explicitly explored the role played by bodily appearance in experiment 3. Previous studies have already shown that non-humanoid shaped objects fail to be integrated and assimilated as pertaining to one's own body. The RHI does not work when a wooden no-hand-shaped object is stroked synchronously with the real hand (Tsakiris and Haggard, 2005). Tsakiris et al. (2010) further extended this finding, pointing out the need for an object to preserve precise, informative corporeal structural features in order to be integrable as one's own body part. Analogously, full body ownership illusions, as well as experimentally induced OBEs do not work when substituting the virtual body with a wooden block having the same dimensions of the fake body (Lenggenhager et al., 2007; Petkova and Ehrsson, 2008). Our experiment went beyond this by testing whether the level of realism of a humanoid body could modulate the intensity of the ownership illusion, and whether it interacts with other factors, such as the provided visuotactile stimulation.

The results from our experiment showed that participants experienced on average significantly higher levels of illusion when seeing a virtual body with common human features in term of skin texture and clothes, compared to the condition in which they were seeing a plastic mannequin. Nevertheless, the view of human-shaped mannequin does not completely dampen the illusion, consistent with previous reports (Petkova and Ehrsson, 2008; Petkova et al., 2011). When compared with findings from previous studies, our results further suggest that there may be an interaction of the body appearance with the visuotactile component. When seeing a mannequin body, synchronous visuotactile stimulation is found to be necessary to induce a vivid full body ownership illusion (Petkova and Ehrsson, 2008; Petkova et al., 2011). At the same time we found that, when the realism of the body is enhanced, a vivid illusion can occur without the need for additional synchronous visuotactile stimuation. Moreover, the illusion was preserved also when incongruent visuotacticle information was processed. This result supports the possibility of a top–down cognitive mechanism that modulates the way in which multisensory information is processed from the bottom–up perceptual stream. Our findings are in fact analogous to what has been established for the RHI: first the hand-object needs to pass a fitness test in terms of anatomical, volumetric and postural constraints (see Tsakiris et al., 2010 and reference therein). Once the fitness test is passed, other features enhancing the realism of the object to incorporate, e.g., the skin texture, can modulate the intensity of the illusion (Haans et al., 2008).

### 4.4. A basic model for the full body ownership illusion

In this section we sketch a speculative basic model for the neural underpinning of the full body ownership illusion on the basis of key findings from neurophysiological studies in the monkey.

We first review the main relevant neurophysiological results that provide a rational for the proposed model. Graziano et al. (2000) reported finding visuoproprioceptive bimodal neurons in the area 5 of the primate parietal cortex, whose properties are extremely relevant for the present discussion. These bimodal neurons respond to both the seen and the perceived (by proprioception) positions of a limb, even when the seen limb is fake. The first important property of such neurons is that they are sensitive to the visual content of the stimuli, so that their response is modulated by the position of an object in their visual RF only if the object contains the proper anatomical features of the limb that the same neurons encode by proprioception. Interestingly, the fake arm used in that study is extremely realistic, having been prepared by a taxidermist from a monkey of the same species. Visual and proprioceptive signals can be additive or may be combined in more complex fashion, according to the specific neuron. However, the overall averaged activity of this bimodal neuronal population shows a modulation associated with the relative positions of the fake and real arms, the activity being the highest when the locations of the two limbs are the closest. The third important property of such neurons is that their response is modulated by the relative position of the fake and real arms only when the fake arm location has a plausible position with respect to the rest of the body, meaning that no modulation was observed when the realistic fake arm was in a non-matching handedness position or in a backward orientation (with the hand being near the shoulder and the cut end extended outward). A further fundamental finding of the same study is the identification of a second population of trimodal neurons (responding to visual, tactile, and proprioceptive signals) in the same area 5. Neurons in this population encode the position of the real arm by proprioception, but not that of a just-seen fake arm; they become sensitive to the view of the fake arm only when the latter is stroked synchronously (but not asynchronously) with the real arm. Area 5 have been also found to host neurons with large RF, which can be bilateral and can involve multiple body parts (Iwamura et al., 1994; Iwamura, 1998), making them plausible candidates for encoding information about the whole body. Other higher-ordered somatosensory areas have been shown to host bimodal and multimodal neuronal populations with large and bilateral RF. These include the ventral intraparietal (VIP) area and a polysensory zone in the precentral gyrus, hosting bimodal visuotactile neurons, as well as trimodal neurons that additionally responds to auditory or vestibular stimuli (Duhamel et al., 1998; Bremmer et al., 2002; Graziano and Cooke, 2006). A more comprehensive review of the neurophysiological studies of body representation in the monkey brain, which are relevant for the study of human body perception is beyond the scope of this paper and can be found in Blanke (2012).

In the following we propose a speculative basic model for the full body ownership illusion, influenced by the numerous analogies between the findings from neurophysiological recording in the monkey discussed above and the results from the work done on experimentally induced bodily illusions, including the main results of the present study. The same model would apply for the RHI. We propose that distinct bimodal and multimodal neuronal populations are responsible for the ownership illusion. A driving population of bimodal visuoproprioceptive neurons, with properties analogous to those of area 5 neurons in the monkey, would yield the minimal contribution necessary for the illusion to occur. Other bimodal and multimodal populations would have instead a secondary modulating effect. The visuoproprioceptive population activates when the seen body looks similar to and is located close to the real hidden body (i.e., when highly congruent static visuoproprioceptive correlations are provided). As for area 5 neurons in the monkey, this population activates only when the virtual body satisfies the main basic anatomical constraints in terms of shape and visual perspective. The higher the spatial coincidence between the virtual and the real body, the more intense would be the driven activity in this population and the resulting sense of ownership. Visuotactile, visual sensorimotor and other multimodal stimulations would trigger other bimodal and/or multimodal populations that have the effect of enhancing or dampening the sense of ownership, depending on whether they are delivered with proper correlations or not.

In this scenario we can isolate various steps for the “building-up” of an ownership illusion, encompassing most of the results that have been found in experimental studies on bodily illusions. If anatomical constraints are not satisfied in terms of body shape and visual perspective, no illusion occurs, because the driving visuoproprioceptive population is shut down, no matter whether other proper multimodal contingencies are provided. When anatomically constraints are satisfied, a high degree of spatial overlap between the virtual body and the real body could enhance the strength of the illusion. The additional contribution of the top–down effect from a high visual realism of the fake body could bring the illusion to saturation. In this condition, the reinforcing modulation of congruent multimodal correlations cannot be appreciated easily, because the illusion is already strong; moreover, the illusion can be sustained despite the dampening effect of incongruent multimodal stimulation (as in experiment 1), although severe and sustained incongruency could break the illusion completely. If anatomically constraints are met but the realism and colocation are not at a high degree, the driving visuoproprioceptive population will be activated, but with a moderate/low intensity that does not necessarily correspond to a perceptual illusion. In this condition, the modulating effects of additional multimodal stimulations can be critical for eliciting a vivid ownership illusion, when congruent, and more effective in breaking the illusion (if present), when incongruent [as in experiment 3, Petkova and Ehrsson (2008), and most of the RHI experiments].

In a recent review, Blanke (2012) has proposed that the changes in self-identification occurring during an OBE could correspond to changes in the size and position of the visual RFs of visuotactile trunk-centered neurons in VIP. Under the effect of synchronous back-stroking, these RFs would extend far enough to encode the fake body that comes to be part of the participant's peripersonal space (Maravita and Iriki, 2004). An additional subpopulation of trimodal neurons integrating visuotactle and vestibular signals would instead be responsible for changes in self-location and visual perspective. Taken together, the basic model we propose here for the full body ownership illusion and the model proposed for OBEs in Blanke (2012), would provide an evidence for the two types of full body illusions to be different perceptual illusions with different underlying patterns of neural activation.

## 5. Conclusions

The aim of our study was to identify the main perceptual cues underlying the full body ownership illusion and to determine their role and mutual interaction in the elicitation of the illusion. We have selectively manipulated visuotactile and visual sensorimotor stimulation, visual perspective, and the appearance of the virtual body.

We have found that having a first person perspective is an essential condition for experiencing the sense of ownership over the virtual body. When 1PP is provided over a realistic virtual body with a high degree of spatial overlap with the real body, the sole effect of congruent visuoproprioceptive cues can provide a sufficient condition for the illusion. In this condition, the additional contribution of congruent visuotactile and/or sensorimotor stimulation is indeed not necessary. Nonetheless, when the degree of spatial overlap (between the real and virtual body) and/or of the visual realism (of the fake body) is not high, congruent multisensory and/or sensorimotor cues are needed to trigger the illusion. In this case, such multimodal cues can be effective in both boosting the illusion, when congruent, and in suppressing it, when incongruent. We furthermore found that, when a high level of illusion is achieved by the synergic merging of various components, incongruent cues can be processed without breaking the illusion and can be perceived as not incorrect.

We discussed these findings showing that they are consistent with most of the previous results on part- and full-body ownership illusions. Motivated by a number of findings from neurophysiological recordings in the macaque monkey, we further propose a speculative basic model for the full body ownership illusion that accounts for most of the published results.

The present work advances our understanding of the critical mechanisms involved in the full body ownership illusion and provides useful implications for research in body-perception, self-consciousness, and numerous virtual reality applications.

### Conflict of interest statement

The authors declare that the research was conducted in the absence of any commercial or financial relationships that could be construed as a potential conflict of interest.

## Footnotes

^1^http://www.gtec.at/Products/Hardware-and-Accessories/g.MOBIlab-Specs-Features

^2^http://www.gtec.at/Products/Software/g.BSanalyze-Specs-Features

^3^http://www.stata.com/stata12/
